# The Role of Perceived Social Support in the Grief Experiences of More Anxious and Self-Compassionate People

**DOI:** 10.1177/00302228241229484

**Published:** 2024-01-24

**Authors:** Ecem Sarper, David L. Rodrigues

**Affiliations:** 1 Centro de Investigação e Intervenção Social (CIS), 56061 Iscte-Instituto Universitário de Lisboa, Lisboa, Portugal

**Keywords:** grief, trait anxiety, self-compassion, perceived social support

## Abstract

Past research showed that high trait anxiety and low self-compassion, along with lack of perceived social support, have been associated with experiencing stronger grief symptoms. However, research is yet to understand *if* and *how* these factors interact among grieving individuals. Results of a cross-sectional study (*N* = 539) showed that perceived social support interacted differently with trait anxiety and self-compassion to shape grief experiences. Unexpectedly, perceived social support did not buffer the association between higher trait anxiety and stronger grief symptoms. Instead, participants with higher trait anxiety reported stronger symptoms only when they perceived to have less social support. In contrast, participants with higher self-compassion reported less symptoms when they perceived to have more social support. These findings show that social support can emphasize the detrimental role of anxiety and the protective role of self-compassion when people are coping with a loss. Implications and suggestions for future research are discussed.

## Introduction

Grief is among the experiences that can foster discomfort in daily life activities (Neimeyer et al., 2021). Although grief symptoms tend to become milder over time for most people (Pan & Liu, 2021), some people struggle with grief for a longer period of time and experience negative grief-related cognitions, decreased emotional regulation, and feelings of preoccupation and anger (Boelen et al., 2006; Pan & Liu, 2021; Vara & Thimm, 2020). In more recent editions of the International Classification of Diseases (ICD-11) and the Diagnostic and Statistical Manual of Mental Disorders (DSM-5-TR), people with these persistent grief experiences can be diagnosed with prolonged grief disorder (Eisma, 2023). And yet, even without this diagnosis, people who are dealing with the loss of a close person are at risk of experiencing negative outcomes. For example, grieving people were found to be at higher risk of having lower sleep quality (Milic et al., 2019), lower life satisfaction (Cooley et al., 2010), and worse subjective well-being (Ott, 2003).

Some researchers have worked to improve the psychological health and well-being of grieving people. This includes developing cognitive-behavioral interventions to help people cope with the loss or understanding the grief experienced after natural disasters. For instance, Sveen et al. (2018) examined differences in grief experiences of survivors following the 2004 Indian Ocean tsunami. The authors found that individuals were more likely to experience grief over time if they had lost a child, reported more post-traumatic stress symptoms, and were less satisfied with the social support they received after the incident. Other researchers took a different approach and examined the correlates of grief experiences among the general population. For example, people with an insecure attachment style (Currier et al., 2015), those who were more emotionally closer to the deceased (Lobb et al., 2010), and those who have problems with substance use (Parisi et al., 2019), are likely to report more severe grief symptoms. However, having more adaptive emotion regulation strategies (e.g., mindfulness) can help decrease the severity of such experiences (Eisma & Stroebe, 2021). Similarly, having stronger support systems can act as a buffer when people are struggling with the loss of a close person (Gesi et al., 2020; Levi-Belz & Lev-Ari, 2019; Mason et al., 2020), and foster personal growth and acceptance after such experience (Hogan & Schmidt, 2002).

Past research has suggested that highly traumatic events can intensify grief experiences, depending on certain individual (e.g., gender; Neria et al., 2007) and relational variables (e.g., negative interpersonal relationships; Burke et al., 2010). However, the interplay between these variables is far less understood. Drawing from the Stress Buffering Hypothesis (Cohen, 2004), social connections can provide psychological resources to help individuals cope with stress in difficult times. Hence, we argue that perceived social support can have a crucial role in determining under which conditions some individuals are *more* or *less* likely to experience severe grief symptoms after the loss of a close person. More specifically, we examined if trait anxiety and self-compassion (i.e., individual factors) were concurrently associated with grief experiences, and whether these associations were differently shaped by perceived social support (i.e., relational factor).

### Individual Determinants of Grief Experiences

Systematic reviews have identified several individual factors that can predispose people to struggle with grief (Dodd et al., 2017; Lobb et al., 2010). Trait anxiety and self-compassion are among the factors that can have the strongest roles in determining how people cope with a loss (Mason et al., 2020; Vara & Thimm, 2020). On the one hand, trait anxiety refers to a type of anxiety that is a relatively stable disposition and tendency (compared to state anxiety), determining the perceptions of dangerousness or riskiness of stimuli (Dolz-del-Castellar & Oliver, 2021; Eysenck, 2000). Trait anxiety has been associated with decreased quality of life, well-being, and physical health over time (Ristvedt & Trinkaus, 2009). On the other hand, self-compassion is an individual predisposition to have an understanding, compassionate, and kind attitude toward oneself when coping with a stressful event (Neff, 2011). People with higher self-compassion enact more health-promoting behaviors and experience fewer negative physical symptoms (Dunne et al., 2018; Jeon et al., 2016).

Research has shown that people with higher trait anxiety tend to report stronger grief symptoms (Piper et al., 2001), possibly because anxious people tend to have poorer coping strategies (Tofthagen et al., 2017) and higher negative metacognitions during stressful events (e.g., beliefs about uncontrollability; Nordahl et al., 2019). In contrast, self-compassion has been shown to protect against severe grief symptoms (Harris, 2021), possibly because people with higher self-compassion tend to be aware of their emotions, have more empathetic attitudes toward themselves in difficult times, and perceive negative events as a part of the human condition (Neff, 2003). Still, results for the positive impact of self-compassion on grief are inconsistent. For example, a recent randomized controlled trial found significant effects of a compassion-based program in reducing grief symptoms (Jahani et al., 2022), whereas another randomized controlled trial found no significant results (Johannsen et al., 2022). And even though trait anxiety and self-compassion are reciprocally and negatively correlated over time (Pagnini et al., 2019), research is yet to determine whether both variables are distinctively and uniquely associated with grief symptoms when considered together. We further argue for the importance of understanding whether the expected associations between both individual factors and grief experiences can be buffered or enhanced by the social support that people perceive to have, when struggling with a loss.

## Relational Determinants of Grief Experiences

Social support has consistently been acknowledged as crucial for physical and psychological outcomes (Reblin & Uchino, 2008; Siedlecki et al., 2014). For example, perceived social support has been associated with greater psychological well-being, including subjective happiness, positive affect, and quality of life, whereas lacking support has been associated with negative affective experiences, including stress and depression (Wilson et al., 2020). Social support offers emotional and instrumental tools for people to cope with difficulties (Bennett et al., 2001) and is beneficial for people struggling with a stressful experience (Scott et al., 2020). For example, research conducted during the initial months of the COVID-19 pandemic has shown that perceived partner support buffered the longitudinal association between experiencing more external stressors and reporting poorer relationship quality (Balzarini et al., 2023), whereas lacking interactions with close others increased the longitudinal association between being more focused on health safety and experiencing more negative affect (Rodrigues et al., 2022).

Specifically focusing on grief, research has shown that having less social support is associated with stronger grief symptoms. For example, people who perceive to have a weaker support system (e.g., lacking understanding or help from close family members) report stronger grief symptoms (Levi-Belz & Lev-Ari, 2019). In contrast, parents who lost a child worked through their grief experiences if they received more support from their partners or close others (Kreicbergs et al., 2007). Likewise, the anticipated grief of family caregivers during palliative care was found to be more strongly related to grief experiences after the loss, but only for caregivers who perceived to have less social support during the process (Axelsson et al., 2020). Moreover, recent studies have shown that greater social support is negatively associated with anxiety (Zhao et al., 2022) and positively associated with self-compassion (Wilson et al., 2020). The benefits of perceived social support on decreased anxiety, increased self-compassion, and fewer severe grief symptoms (Levi-Belz & Lev-Ari, 2019) arguably suggest a regulatory function of perceived social support, and highlight the importance of examining under which conditions and for whom perceived social support is crucial when coping with grief.

### Current Study and Hypotheses

In a cross-sectional study with a sample of adults who experienced the loss of a close person, we examined if the associations between individual factors (i.e., trait anxiety and self-compassion) and grief symptoms were moderated by perceived social support (i.e., a relational factor). Building upon past research, we expected more trait anxiety (H1), less self-compassion (H2), and less social support (H3) to be associated with stronger grief symptoms. We also expected perceived social support to moderate the associations of both individual factors with stronger grief symptoms (H4). Specifically, we expected perceived social support to buffer the positive association between trait anxiety and grief symptoms (H4a), and to enhance the negative association between self-compassion and grief symptoms (H4b). Given that emotional intimacy with the deceased (Lobb et al., 2010) and having lost this person more recently (Pan & Liu, 2021) tend to intensify negative feelings during the grieving process, both variables were controlled for in our analyses.

## Method

### Participants and Sampling

Prospective participants were recruited from European countries (e.g., Portugal, Cyprus, Turkey, the UK), either through the Clickworker recruitment platform, by sharing the link to the online survey in different outlets (e.g., Instagram; Sapo Lifestyle), or through the participant pool at Iscte-Instituto Universitário de Lisboa. Participation was restricted to people who were over 18 years of age and experienced the loss of a close person (e.g., a family member, a friend, or a romantic partner). From the 897 people who accessed the online survey, we excluded those who did not experience the loss of a close person (*n* = 237), those who failed to complete the survey (*n* = 111), and those who failed to complete the variables under examination (*n* = 10). Participants from the final sample (*N* = 539) were, on average, 34 years old, and most identified as female, completed more than 12 years of education, and were struggling to live or managing on their current income. Most participants indicated having lost their grandparents or parents, and most reported chronic disease as the cause of death. Sample characteristics can be found in Table 1.

**Table 1.** Sociodemographic Characteristics.

	% (*n*)	*M* (*SD*)
Age (range: 18–81 years)		33.5 (11.4)
Gender		
Female	57.1 (308)	
Male	42.5 (229)	
Other (e.g., nonbinary)	0.4 (2)	
Education		
Less than or equal to 12 years	29.9 (161)	
More than 12 years	70.1 (378)	
Perceived socioeconomic status		
Difficult to live on current income	42.3 (228)	
Managing on current income	43.0 (232)	
Living comfortably on current income	14.7 (79)	
Relationship with the deceased		
Mother/father	27.8 (150)	
Child	1.5 (8)	
Sibling	3.2 (17)	
Romantic partner	5.9 (32)	
Close friend	19.9 (107)	
Grandparents	28.1 (149)	
Other relatives (e.g., aunt, uncle, cousin)	13.6 (76)	
Cause of death		
Chronic disease	38.0 (205)	
Sudden health failure	25.2 (136)	
COVID-19	8.2 (44)	
Homicide	1.5 (8)	
Suicide	4.6 (25)	
Traffic-related accident	11.5 (62)	
Health-related causes	6.5 (35)	
Non-traffic accidents and natural disasters	2.4 (13)	
Aging	1.3 (7)	
Missing responses	0.7 (4)	

### Measures

Our main outcome variables (i.e., trait anxiety, self-compassion, perceived social support, and grief symptoms) were assessed with measures previously validated in different cultural contexts. As we recruited an international sample of participants, we had to assure the overall construct validity of these measures. Hence, we computed Exploratory Factor Analyses (EFA) with principal axis factoring and promax rotation on our sample. Items with loadings >.30 in more than one factor and those with loadings <.30 in all factors were removed until a final solution was reached for each scale. We then examined the internal reliability of the final factor structures.

#### Demographic Information

Participants were asked to indicate standard demographic information including age (open-ended question), gender (1 = *Female*, 2 = *Male*, 3 = *Other, please specify* [open-ended]), education level (1 = *Less than or equal to 12 years of education*, 2 = *More than 12 years of education*), perceived socioeconomic status (1 = *Finding it difficult to live on my current income*, 2 = *Managing to live with my* *current income*, 3 = *Living comfortably on my current income*).

#### Loss-Related Information, Emotional Intimacy, and Time Since the Loss

Participants were asked to indicate the relationship they had with the person they lost (“What was your relationship with the person who died?”; 1 = *Mother/father*, 2 = *Child*, 3 = *Sibling*, 4 = *Romantic partner*, 5 = *Close friend*, 6 = *Grandparents*, 7 = *Other relatives, please specify* [open ended]), the cause of death of this person (“What was the cause of their death?”; 1 = *Chronic disease*, 2 = *Sudden health failure*, 3 = *COVID-19*, 4 = *Homicide*, 5 = *Suicide*, 6 = *Traffic-related accident*, 7 = *Other, please specify* [open ended]) and the emotional intimacy they had with this person (“How would you define the emotional intimacy between you and the most significant person you lost?”; from 1 = *Not Intimate at all* to 5 = *Very Intimate*). Higher values in the last item indicate more emotional intimacy with the deceased (*M* = 4.39, *SD* = 0.71). Participants were additionally asked to write down (open-ended question) how much time has passed since their loss (“When did you experience this loss? [Please write down the month and the year of your experience]”). Responses were recoded into months (*M* = 55.35, *SD* = 76.83). Participants who indicated they lost more than one person were asked to focus on the loss they considered to be the most significant.

#### Trait Anxiety

We used the 20-item Trait Anxiety Subscale of the State-Trait Anxiety Inventory (Spielberger, 1983). Participants were asked to report how they generally feel (e.g., “I feel nervous and restless.”) using 4-point rating scales (1 = *Almost Never* to 4 = *Almost Always*). EFA results yielded a 15-item scale with a one-factor structure that explained 43.12% of the total variance. Responses were mean averaged into a single score (*α* = .92; *M* = 2.41, *SD* = 0.53), with higher scores indicating higher trait anxiety.

#### Self-Compassion

We used the 12-item Self-Compassion Scale – Short Form (Raes et al., 2011) and asked participants to indicate how they think about themselves (e.g., “I try to see my failings as part of the human condition.”). Responses were given in 5-point rating scales (1 = *Almost Never* to 5 = *Almost Always*). Similar to the original scale, EFA results supported a 12-item scale with a one-factor structure that explained 40.53% of the total variance. Responses were mean averaged in a single score (*α* = .88; *M* = 3.13*, SD* = 0.78), with higher scores indicating higher self-compassion.

#### Perceived Social Support

Using the 12-item Multidimensional Scale of Perceived Social Support (Zimet et al., 1988), we asked participants to indicate their level of support from close others (e.g., “I have friends with whom I can share my joys and sorrows.”) using 7-point rating scales (1 = *Very Strongly Disagree* to 7 = *Very Strongly Agree*). As in the original scale, EFA results supported a 12-item scale with a one-factor structure that explained 49.93% of the total variance. Responses were mean averaged in a single index (*α* = .92; *M* = 5.16*, SD* = 1.42), with higher scores indicating higher perceived social support.

#### Grief Experiences

We used the 19-item Inventory of Complicated Grief (Prigerson et al., 1995) to assess grief experiences following the loss of a close person. Participants were asked to indicate their feelings and reactions thinking about their most significant loss (e.g., “I feel myself longing for the person who died.”) using 5-point rating scales (0 = *Never* to 4 = *Always*). EFA results yielded a 13-item scale with a one-factor structure that explained 34.98% of the total variance. Responses were mean averaged into a single index (*α* = .87; *M* = 1.27, *SD* = 0.72), with higher scores indicating stronger grief symptoms.

### Procedure

This study was approved by the Ethics Council at Iscte-Instituto Universitário de Lisboa (#22/2021). The survey was available in three different languages: Turkish (*n* = 258), Portuguese (*n* = 228), and English (*n* = 53). When accessing the link to the online study, prospective participants were informed about the potential distressing effects of some questions included in the survey, and their right to abandon the study without justification or penalty. Participants could only proceed to the survey after providing their consent. After answering standard demographic questions (e.g., age, gender), participants were presented with the remaining measures. In the end, participants were thanked for their collaboration, explained the goals of the study, and offered contacts for public services in case they wanted to seek out professional help. This study was part of a larger project aimed at examining different implications of grief and included other measures not relevant to the current analyses.

### Data Analytic Plan

We first computed overall correlations between our main measures and covariates. Our hypotheses were tested using a linear regression model. We entered scores for trait anxiety, self-compassion, perceived social support, as well as the interactions between both individual factors and perceived social support. We also entered emotional intimacy and time since loss as covariates in the model. All variables were standardized before the analysis and before the interaction terms (Aiken et al., 1991). When significant interactions were found, we computed and plotted simple slopes for participants who perceived less (−1 *SD*) or more (+1 *SD*) social support (Preacher et al., 2006).

## Results

### Preliminary Analysis

Descriptive statistics and overall correlations between our main variables and covariates are summarized in Table 2. As can be seen, higher trait anxiety was associated with lower self-compassion, *p* < .001, lower perceived social support, *p* < .001, and stronger grief symptoms, *p* < .001. In contrast, higher self-compassion was associated with higher perceived social support, *p* < .001, and fewer grief symptoms, *p* < .001. Likewise, perceiving more social support was associated with fewer grief symptoms, *p* < .001. Lastly, having more emotional intimacy with the deceased, *p* < .001, and having experienced the loss more recently, *p* < .010, were both associated with stronger grief symptoms.

**Table 2.** Overall Descriptive Statistics and Correlations Between Meas*ures*.

	*M* (*SD*)	Correlations				
		1	2	3	4	5
1. Trait anxiety	2.41 (0.53)	-				
2. Self-compassion	3.13 (0.78)	−.82***	-			
3. Perceived social support	5.16 (1.42)	−.36***	.30***	-		
4. Grief symptoms	1.27 (0.72)	.37***	−.33***	−.16***	-	
5. Emotional intimacy with the deceased	4.39 (0.71)	−.01	−.01	.05	.30***	-
6. Time since the loss	55.35 (76.83)	−.11*	.04	.02	−.11**	.06

*p ≤ .050,

**p ≤ .010,

***p ≤ .001.

### Social Support in Grief Experiences

As expected, results of the linear regression (see Table 3) showed that higher trait anxiety, *p* < .001 (H1), higher emotional intimacy with the deceased, *p* < .001, and less time since the loss, *p* = .011 (both covariates), were associated with stronger grief symptoms. Unexpectedly, no direct associations with grief experiences emerged for self-compassion, *p* = .277 (H2), or perceived social support, *p* = .482 (H3).

**Table 3.** Linear Regression and Simple Slopes.

	*b* (*SE*)
Trait anxiety	.29*** (.07)
Self-compassion	−.07 (.07)
Perceived social support	−.03 (.04)
Trait anxiety x perceived social support	−.16* (.06)
Less perceived support (−1 *SD*)	.45*** (.09)
More perceived support (+1 *SD*)	.14 (.09)
Self-compassion x perceived social support	−.14* (.06)
Less perceived support (−1 *SD*)	.06 (.09)
More perceived support (+1 *SD*)	−.21* (.09)
Emotional intimacy with the deceased (Cov.)	.30*** (.04)
Time since the loss (Cov.)	−.10* (.04)
Adjusted *R*^*2*^	.24***

*Note.* All variables were standardized before the analyses. Results revealed the absence of collinearity between predictors, with tolerance values ≥0.31 and Variance Inflation Factor (VIF) ≤ 3.28.

*p ≤ .050.

**p ≤ .010.

***p ≤ .001.

**Figure 1.**
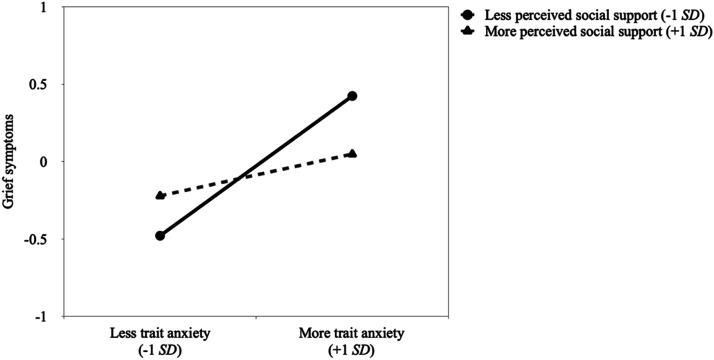
Association between trait anxiety and grief symptoms at lower (-1*SD*) and higher (+1*SD*) values of perceived social support.

More importantly, results showed the expected significant interactions between trait anxiety and perceived social support, *p* = .011, and between self-compassion and perceived social support, *p* = .024 (H4). Contrary to our buffer hypothesis (H4a), simple slope analyses revealed that participants with higher (vs. lower) trait anxiety reported stronger grief symptoms if they perceived to have less social support, *p* < .001, but not more social support, *p* = .146 (see Figure 1). Additional comparisons showed that participants who perceived to have less social support reported stronger grief symptoms if they had higher (vs. lower) trait anxiety, *p* = .006. In contrast, participants who perceived to have more social support did not differ in their grief symptoms according to trait anxiety levels, *p* = .116.

**Figure 2.**
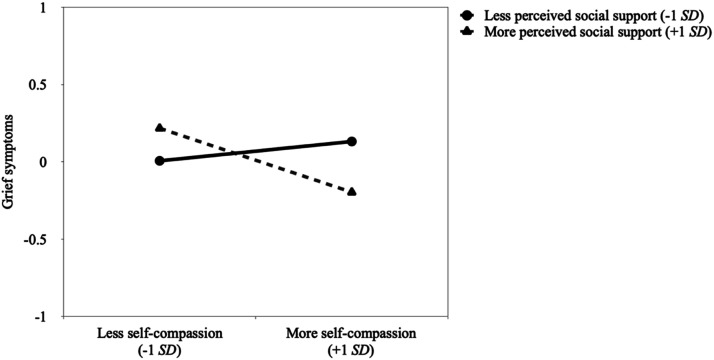
Association between self-compassion and grief symptoms at lower (-1*SD*) and higher (+1*SD*) values of perceived social support.

Aligned with our enhancement hypothesis (H4b), simple slope analyses revealed that participants with higher (vs. lower) self-compassion reported fewer grief symptoms if they perceived to have more social support, *p* = .025, but not less social support, *p* = .467 (see Figure 2). Additional comparisons showed that participants who perceived to have less social support did not differ in their grief symptoms according to self-compassion levels, *p* = .160. In contrast, participants who perceived to have more social support reported weaker grief symptoms if they had higher (vs. lower) self-compassion, *p* = .019.

## Discussion

We examined whether trait anxiety and self-compassion were associated with grief experiences among people coping with the loss of a close person, and if perceived social support was a condition under which these associations changed. Results partially supported our hypotheses. When considered separately, individual and relational factors were correlated with grief experiences, such that more trait anxiety, less self-compassion, and less perceived social support were associated with stronger grief symptoms. When considered concurrently, however, the relative contribution of each individual factor to grief experiences differed. Specifically, trait anxiety contributed to stronger grief symptoms (H1), whereas the contributions of self-compassion (H2) and perceived social support (H3) were rendered non-significant. Past research has already shown that people who are anxious consolidate negative memories more easily and show a cognitive bias when recalling negative events (Wang et al., 2019). Our results suggest this may be particularly relevant when people are coping with the loss of a close person, regardless of their predisposition to be compassionate about themselves, or the level of social support they receive from other people in their close network. Our non-significant result for self-compassion is aligned with findings from a recent randomized controlled trial (Johannsen et al., 2022).

We also found mixed evidence regarding the beneficial role of perceived social support. As expected, perceived social support moderated the association between each individual factor and grief experiences (H4). However, perceiving more social support did not buffer the association between higher trait anxiety and stronger grief symptoms (H4a). Instead, *lacking* perceived social support emphasized the grief symptoms of participants who reported higher trait anxiety. Congruent with the negative cognitive bias explanation (Wang et al., 2019), perceiving to be less supported by close others during such a difficult time can make negative grief-related memories even more salient and result in the experience of stronger grief symptoms. In contrast, and as expected, perceiving to have more social support enhanced the protective role of self-compassion against grief symptoms (H4b). This finding is aligned with the Stress Buffering Hypothesis (Cohen, 2004), showing that having a supportive close network has benefits for health and well-being. More specific to grief experiences, our results are aligned with past research (Ogrodniczuk et al., 2002) and suggest that perceived social support from close others can have different regulatory functions for people who vary in certain personality traits. According to our results, however, this benefit seems to occur only when grieving people already have a predisposition to have a more understanding frame of mind and feel compassionate about themselves. In other words, people with lower trait anxiety and lower self-compassion may be more likely to cope with grief in a way that contextual variables are not determinant over and above their individual variables or personality traits. For people with higher trait anxiety and higher self-compassion, on the other hand, this social context seems to matter by having consequences (in the former case) and benefits (in the latter case) for the grieving process.

### Limitations and Future Research

This study has some limitations that need to be acknowledged. First, our data is correlational, which prevents us from establishing causality. Even though we reasoned that individual factors precede and contribute to how people react and grieve the loss of a close person, it may also be that the trauma of losing a close person leads people to become more anxious and less self-compassionate in their daily lives. Future studies could consider conducting longitudinal studies and whenever possible, in more ecologically valid contexts (e.g., after a natural disaster, much like the recent earthquake that happened in Turkey and Syria in February 2023). Moreover, researchers could establish baseline scores for risk factors associated with stronger grief symptoms in non-grieving cohorts, including trait anxiety and self-compassion, to address potential confounds between individual traits and the grief event. We also used a general measure of perceived social support and were unable to determine whether the perceived or actual support received from different sources (e.g., close friends vs. close family members vs. romantic partner) contributed differently when people are coping with grief. For example, research has shown that social support from online communities can decrease feelings of loneliness (Fogel et al., 2002) and improve subjective well-being (Wangberg et al., 2008) for people coping with stressful events (e.g., receiving a cancer treatment). Examining longitudinal associations, establishing baseline scores in individual traits, and considering different sources of perceived and actual social support would allow researchers to replicate and extend our findings by examining the natural progression of the grieving process, if people who are more anxious and less self-compassionate have greater risk of developing stronger grief symptoms over time, and if social support from multiple sources (e.g., close others, network communities, social structures) can worsen or ameliorate the grieving process.

## Conclusion

Our study shows the detriments and benefits of individual and relational factors among a community sample of people who have been affected by a loss experience. Our findings highlight the importance of assessing multiple individual factors when helping people cope with a loss and shed light on who is at greater risk of experiencing stronger grief symptoms. From a theoretical perspective, our findings complement past research examining individual determinants of grief experiences (Diolaiuti et al., 2021; Glad et al., 2022; Goetter et al., 2019; Vara & Thimm, 2020). More importantly, as findings clearly show the crucial role of social support during the grieving process, we add to the theoretical discussion the interplay between individual and relational determinants of increased grief-related symptoms. From an applied perspective, our findings can offer insights to develop protocols and interventions adjusted to the personal needs and conditions people are in (Iglewicz et al., 2020; Mason et al., 2020), aiming to decrease the severity of grief symptoms and increase the subjective well-being and health outcomes of people coping with a loss.

## References

[bibr1-00302228241229484] Aiken L. S. West S. G. Reno R. R. (1991). Multiple regression: Testing and interpreting interactions. Sage Publications.

[bibr3-00302228241229484] Axelsson L. Alvariza A. Holm M. Årestedt K. (2020). Intensity of predeath grief and postdeath grief of family caregivers in palliative care in relation to preparedness for caregiving, caregiver burden, and social support. Palliative Medicine Reports, 1(1), 191–200. 10.1089/pmr.2020.0033 34223476 PMC8241336

[bibr4-00302228241229484] Balzarini R. N. Muise A. Zoppolat G. Di Bartolomeo A. Rodrigues D. L. Alonso-Ferres M. Urganci B. Debrot A. Bock Pichayayothin N. Dharma C. Chi P. Karremans J. C. Schoebi D. Slatcher R. B. (2023). Love in the time of COVID: Perceived partner responsiveness buffers people from lower relationship quality associated with COVID-related stressors. Social Psychological and Personality Science, 14(3), 342–355. 10.1177/19485506221094437

[bibr5-00302228241229484] Bennett S. J. Perkins S. M. Lane K. A. Deer M. Brater D. C. Murray M. D. (2001). Social support and health-related quality of life in chronic heart failure patients. Quality of Life Research, 10(8), 671–682. 10.1023/A:1013815825500 11871588

[bibr7-00302228241229484] Boelen P. A. van den Hout M. A. van den Bout J. (2006). A cognitive-behavioral conceptualization of complicated grief. Clinical Psychology: Science and Practice, 13(2), 109–128. 10.1111/j.1468-2850.2006.00013.x

[bibr8-00302228241229484] Burke L. A. Neimeyer R. A. McDevitt-Murphy M. E. (2010). African American homicide bereavement: Aspects of social support that predict complicated grief, PTSD, and depression. OMEGA - Journal of Death and Dying, 61(1), 1–24. 10.2190/OM.61.1.a 20533646

[bibr9-00302228241229484] Cohen S. (2004). Social relationships and health. American Psychologist, 59(8), 676–684. 10.1037/0003-066X.59.8.676 15554821

[bibr10-00302228241229484] Cooley E. Toray T. Roscoe L. (2010). Reactions to loss scale: Assessing grief in college students. OMEGA - Journal of Death and Dying, 61(1), 25–51. 10.2190/OM.61.1.b 20533647

[bibr11-00302228241229484] Currier J. M. Irish J. E. F. Neimeyer R. A. Foster J. D. (2015). Attachment, continuing bonds, and complicated grief following violent loss: Testing a moderated model. Death Studies, 39(4), 201–210. 10.1080/07481187.2014.975869 25551174

[bibr12-00302228241229484] Diolaiuti F. Marazziti D. Beatino M. F. Mucci F. Pozza A. (2021). Impact and consequences of COVID-19 pandemic on complicated grief and persistent complex bereavement disorder. Psychiatry Research, 300, 113916. 10.1016/j.psychres.2021.113916 33836468 PMC8479443

[bibr13-00302228241229484] Dodd A. Guerin S. Delaney S. Dodd P. (2017). Complicated grief: Knowledge, attitudes, skills and training of mental health professionals: A systematic review. Patient Education and Counseling, 100(8), 1447–1458. 10.1016/j.pec.2017.03.010 28320560

[bibr14-00302228241229484] Dolz-del-Castellar B. Oliver J. (2021). Relationship between family functioning, differentiation of self and anxiety in Spanish young adults. PLoS One, 16(3), Article e0246875. 10.1371/journal.pone.0246875 33657141 PMC7928452

[bibr15-00302228241229484] Dunne S. Sheffield D. Chilcot J. (2018). Brief report: Self-compassion, physical health and the mediating role of health-promoting behaviours. Journal of Health Psychology, 23(7), 993–999. 10.1177/1359105316643377 27121978

[bibr16-00302228241229484] Eisma M. C. (2023). Prolonged grief disorder in ICD-11 and *DSM* -5-TR: Challenges and controversies. Australian and New Zealand Journal of Psychiatry, 57(7), 944–951. 10.1177/00048674231154206 36748103 PMC10291380

[bibr17-00302228241229484] Eisma M. C. Stroebe M. S. (2021). Emotion regulatory strategies in complicated grief: A systematic review. Behavior Therapy, 52(1), 234–249. 10.1016/j.beth.2020.04.004 33483120

[bibr18-00302228241229484] Eysenck M. W. (2000). A cognitive approach to trait anxiety. European Journal of Personality, 14(5), 463–476. 10.1002/1099-0984(200009/10)14:5<463::AID-PER393>3.0.CO

[bibr19-00302228241229484] Fogel J. Albert S. M. Schnabel F. Ditkoff B. A. Neugut A. I. (2002). Internet use and social support in women with breast cancer. Health Psychology, 21(4), 398–404. 10.1037/0278-6133.21.4.398 12090683

[bibr20-00302228241229484] Gesi C. Carmassi C. Cerveri G. Carpita B. Cremone I. M. Dell’Osso L. (2020). Complicated grief: What to expect after the coronavirus pandemic. Frontiers in Psychiatry, 11, 489. 10.3389/fpsyt.2020.00489 32574243 PMC7264152

[bibr21-00302228241229484] Glad K. A. Stensland S. Czajkowski N. O. Boelen P. A. Dyb G. (2022). The longitudinal association between symptoms of posttraumatic stress and complicated grief: A random intercepts cross-lag analysis. Psychological Trauma: Theory, Research, Practice, and Policy, 14(3), 386–392. 10.1037/tra0001087 34398627

[bibr22-00302228241229484] Goetter E. Bui E. Horenstein A. Baker A. W. Hoeppner S. Charney M. Simon N. M. (2019). Five-factor model in bereaved adults with and without complicated grief. Death Studies, 43(3), 204–209. 10.1080/07481187.2018.1446059 29498608

[bibr23-00302228241229484] Harris D. (2021). Compassion-focused grief therapy. British Journal of Guidance and Counselling, 49(6), 780–790. 10.1080/03069885.2021.1960948

[bibr24-00302228241229484] Hogan N. S. Schmidt L. A. (2002). Testing the grief to personal growth model using structural equation modeling. Death Studies, 26(8), 615–634. 10.1080/07481180290088338 12243195

[bibr25-00302228241229484] Iglewicz A. Shear M. K. Reynolds C. F. Simon N. Lebowitz B. Zisook S. (2020). Complicated grief therapy for clinicians: An evidence‐based protocol for mental health practice. Depression and Anxiety, 37(1), 90–98. 10.1002/da.22965 31622522

[bibr26-00302228241229484] Jahani L. Abolhassani S. Babaee S. Omranifard V. (2022). Effects of a compassion-based program on the grief experienced by caregivers of people suffering from dementia: A randomized controlled clinical trial. BMC Nursing, 21(1), 198. 10.1186/s12912-022-00980-5 35879751 PMC9316726

[bibr27-00302228241229484] Jeon H. Lee K. Kwon S. (2016). Investigation of the structural relationships between social support, self-compassion, and subjective well-being in Korean elite student athletes. Psychological Reports, 119(1), 39–54. 10.1177/0033294116658226 27381414

[bibr28-00302228241229484] Johannsen M. Schlander C. Farver-Vestergaard I. Lundorff M. Wellnitz K. B. Komischke-Konnerup K. B. O’Connor M. (2022). Group-based compassion-focused therapy for prolonged grief symptoms in adults – results from a randomized controlled trial. Psychiatry Research, 314, 114683. 10.1016/j.psychres.2022.114683 35717855

[bibr29-00302228241229484] Kreicbergs U. C. Lannen P. Onelov E. Wolfe J. (2007). Parental grief after losing a child to cancer: Impact of professional and social support on long-term outcomes. Journal of Clinical Oncology, 25(22), 3307–3312. 10.1200/JCO.2006.10.0743 17664479

[bibr31-00302228241229484] Levi-Belz Y. Lev-Ari L. (2019). Is there anybody out there? Attachment style and interpersonal facilitators as protective factors against complicated grief among suicide-loss survivors. The Journal of Nervous and Mental Disease, 207(3), 131–136. 10.1097/NMD.0000000000000940 30720603

[bibr32-00302228241229484] Lobb E. A. Kristjanson L. J. Aoun S. M. Monterosso L. Halkett G. K. B. Davies A. (2010). Predictors of complicated grief: A systematic review of empirical studies. Death Studies, 34(8), 673–698. 10.1080/07481187.2010.496686 24482845

[bibr33-00302228241229484] Mason T. M. Tofthagen C. S. Buck H. G. (2020). Complicated grief: Risk factors, protective factors, and interventions. Journal of Social Work in End-of-Life and Palliative Care, 16(2), 151–174. 10.1080/15524256.2020.1745726 32233740

[bibr34-00302228241229484] Milic J. Saavedra Perez H. Zuurbier L. A. Boelen P. A. Rietjens J. A. Hofman A. Tiemeier H. (2019). The longitudinal and cross-sectional associations of grief and complicated grief with sleep quality in older adults. Behavioral Sleep Medicine, 17(1), 31–40. 10.1080/15402002.2016.1276016 28107032

[bibr35-00302228241229484] Neff K. (2003). Self-compassion: An alternative conceptualization of a healthy attitude toward oneself. Self and Identity, 2(2), 85–101. 10.1080/15298860309032

[bibr36-00302228241229484] Neff K. D. (2011). Self-compassion, self-esteem, and well-being: Self-compassion, self-esteem, and well-being. Social and Personality Psychology Compass, 5(1), 1–12. 10.1111/j.1751-9004.2010.00330.x

[bibr37-00302228241229484] Neimeyer R. A. Testoni I. Ronconi L. Biancalani G. Antonellini M. Dal Corso L. (2021). The integration of stressful life experiences scale and the inventory of complicated spiritual grief: The Italian validation of two instruments for meaning-focused assessments of bereavement. Behavioral Sciences, 11(11), 149. 10.3390/bs11110149 34821610 PMC8614745

[bibr38-00302228241229484] Neria Y. Gross R. Litz B. Maguen S. Insel B. Seirmarco G. Rosenfeld H. Suh E. J. Kishon R. Cook J. Marshall R. D. (2007). Prevalence and psychological correlates of complicated grief among bereaved adults 2.5–3.5 years after September 11th attacks. Journal of Traumatic Stress, 20(3), 251–262. 10.1002/jts.20223 17597124

[bibr39-00302228241229484] Nordahl H. Hjemdal O. Hagen R. Nordahl H. M. Wells A. (2019). What lies beneath trait-anxiety? Testing the self-regulatory executive function model of vulnerability. Frontiers in Psychology, 10, 122. 10.3389/fpsyg.2019.00122 30804834 PMC6371045

[bibr40-00302228241229484] Ogrodniczuk J. S. Piper W. E. Joyce A. S. McCallum M. Rosie J. S. (2002). Social support as a predictor of response to group therapy for complicated grief. Psychiatry: Interpersonal and Biological Processes, 65(4), 346–357. 10.1521/psyc.65.4.346.20236 12530338

[bibr41-00302228241229484] Ott C. H. (2003). The impact of complicated grief on mental and physical health at various points in the bereavement process. Death Studies, 27(3), 249–272. 10.1080/07481180302887 12703505

[bibr42-00302228241229484] Pagnini F. Cavalera C. Rovaris M. Mendozzi L. Molinari E. Phillips D. Langer E. (2019). Longitudinal associations between mindfulness and well-being in people with multiple sclerosis. International Journal of Clinical and Health Psychology, 19(1), 22–30. 10.1016/j.ijchp.2018.11.003 30619494 PMC6300715

[bibr43-00302228241229484] Pan H. Liu F. (2021). The prevalence of complicated grief among Chinese people at high risk: A systematic review and meta-analysis. Death Studies, 45(6), 480–490. 10.1080/07481187.2019.1648342 31402787

[bibr44-00302228241229484] Parisi A. Sharma A. Howard M. O. Blank Wilson A. (2019). The relationship between substance misuse and complicated grief: A systematic review. Journal of Substance Abuse Treatment, 103, 43–57. 10.1016/j.jsat.2019.05.012 31229191

[bibr45-00302228241229484] Piper W. E. Ogrodniczuk J. S. Azim H. F. Weideman R. (2001). Prevalence of loss and complicated grief among psychiatric outpatients. Psychiatric Services, 52(8), 1069–1074. 10.1176/appi.ps.52.8.1069 11474053

[bibr46-00302228241229484] Preacher K. J. Curran P. J. Bauer D. J. (2006). Computational tools for probing interactions in multiple linear regression, multilevel modeling, and latent curve analysis. Journal of Educational and Behavioral Statistics, 31(4), 437–448. 10.3102/10769986031004437

[bibr47-00302228241229484] Prigerson H. G. Maciejewski P. K. Reynolds C. F. Bierhals A. J. Newsom J. T. Fasiczka A. Frank E. Doman J. Miller M. (1995). Inventory of complicated grief: A scale to measure maladaptive symptoms of loss. Psychiatry Research, 59(1–2), 65–79. 10.1016/0165-1781(95)02757-2 8771222

[bibr48-00302228241229484] Raes F. Pommier E. Neff K. D. Van Gucht D. (2011). Construction and factorial validation of a short form of the Self-Compassion Scale. Clinical Psychology and Psychotherapy, 18(3), 250–255. 10.1002/cpp.702 21584907

[bibr49-00302228241229484] Reblin M. Uchino B. N. (2008). Social and emotional support and its implication for health. Current Opinion in Psychiatry, 21(2), 201–205. 10.1097/YCO.0b013e3282f3ad89 18332671 PMC2729718

[bibr50-00302228241229484] Ristvedt S. L. Trinkaus K. M. (2009). Trait anxiety as an independent predictor of poor health-related quality of life and post-traumatic stress symptoms in rectal cancer. British Journal of Health Psychology, 14(4), 701–715. 10.1348/135910708X400462 19171084 PMC2756319

[bibr51-00302228241229484] Rodrigues D. L. Zoppolat G. Balzarini R. N. Slatcher B.R. (2022). Security motives and negative affective experiences during the early months of the COVID-19 pandemic. Psychology and Health, 37(12), 1605–1625. 10.1080/08870446.2022.2067332 35510649

[bibr52-00302228241229484] Scott H. R. Pitman A. Kozhuharova P. Lloyd-Evans B. (2020). A systematic review of studies describing the influence of informal social support on psychological wellbeing in people bereaved by sudden or violent causes of death. BMC Psychiatry, 20(1), 265. 10.1186/s12888-020-02639-4 32471407 PMC7257446

[bibr53-00302228241229484] Siedlecki K. L. Salthouse T. A. Oishi S. Jeswani S. (2014). The relationship between social support and subjective well-being across age. Social Indicators Research, 117(2), 561–576. 10.1007/s11205-013-0361-4 25045200 PMC4102493

[bibr54-00302228241229484] Spielberger C. D. (1983). State-trait anxiety inventory for adults [dataset]. American Psychological Association. 10.1037/t06496-000

[bibr55-00302228241229484] Sveen J. Bergh Johannesson K. Cernvall M. Arnberg F. K. (2018). Trajectories of prolonged grief one to six years after a natural disaster. PLoS One, 13(12), Article e0209757. 10.1371/journal.pone.0209757 30576369 PMC6303052

[bibr56-00302228241229484] Tofthagen C. Kip K. Witt A. McMillan S. (2017). Complicated grief: Risk factors, interventions, and resources for oncology nurses. Clinical Journal of Oncology Nursing, 21(3), 331–337. 10.1188/17.CJON.331-337 28524889

[bibr58-00302228241229484] Vara H. Thimm J. C. (2020). Associations between self-compassion and complicated grief symptoms in bereaved individuals: An exploratory study. Nordic Psychology, 72(3), 235–247. 10.1080/19012276.2019.1684347

[bibr59-00302228241229484] Wang T. Li M. Xu S. Liu B. Wu T. Lu F. Xie J. Peng L. Wang J. (2019). Relations between trait anxiety and depression: A mediated moderation model. Journal of Affective Disorders, 244, 217–222. 10.1016/j.jad.2018.09.074 30359817

[bibr60-00302228241229484] Wangberg S. C. Andreassen H. K. Prokosch H.-U. Santana S. M. V. Sorensen T. Chronaki C. E. (2008). Relations between Internet use, socio-economic status (SES), social support and subjective health. Health Promotion International, 23(1), 70–77. 10.1093/heapro/dam039 18083686

[bibr61-00302228241229484] Wilson J. M. Weiss A. Shook N. J. (2020). Mindfulness, self-compassion, and savoring: Factors that explain the relation between perceived social support and well-being. Personality and Individual Differences, 152, 109568. 10.1016/j.paid.2019.109568

[bibr62-00302228241229484] Zhao G. Xie F. Li S. Ding Y. Li X. Liu H. (2022). The relationship between perceived social support with anxiety, depression, and insomnia among Chinese college students during the COVID-19 pandemic: The mediating role of self-control. Frontiers in Psychiatry, 13, 994376. 10.3389/fpsyt.2022.994376 36276317 PMC9582516

[bibr63-00302228241229484] Zimet G. D. Dahlem N. W. Zimet S. G. Farley G. K. (1988). The multidimensional scale of perceived social support. Journal of Personality Assessment, 52(1), 30–41. 10.1207/s15327752jpa5201_2 2280326

